# Ca*_n_* neutral clusters: a two-step *G*_0_*W*_0_ and DFT benchmark

**DOI:** 10.3762/bjnano.15.82

**Published:** 2024-08-08

**Authors:** Sunila Bakhsh, Sameen Aslam, Muhammad Khalid, Muhammad Sohail, Sundas Zafar, Sumayya Abdul Wadood, Kareem Morsy, Muhammad Aamir Iqbal

**Affiliations:** 1 Department of Physics, Balochistan University of Information Technology Engineering and Management Sciences, Quetta 87300, Pakistan; 2 Department of Physics, University of Balochistan, Quetta 87300, Pakistan; 3 Department of Physics, Sardar Bahadur Khan Women University, Quetta 87300, Pakistan; 4 Biology Department, College of Science, King Khalid University, Abha 61421, Saudi Arabia; 5 School of Materials Science and Engineering, Zhejiang University, Hangzhou 310027, China

**Keywords:** calcium clusters, density functional theory, *G*_0_*W*_0_ approximation, ionization potentials, magic clusters

## Abstract

Electronic and structural properties of calcium clusters with a varying size range of 2–20 atoms are studied using a two-step scheme within the *GW* and density functional theory (DFT) with generalized gradient approximation (GGA). The GGA overestimates the binding energies, optimized geometries, electron affinities, and ionization potentials reported in the benchmark. The ground-state structure geometry and binding energy were obtained from the DFT for the ground-state structure of each cluster. The binding energy of the neutral clusters of the calcium series follows an increasing trend, except for a few stable even and odd clusters. The electronic properties of the calcium cluster were studied with an all-electron FHI-aims code. In the *G*_0_*W*_0_ calculation, the magic cluster Ca_10_ has relatively high ionization potential and low electron affinity. The obtained ionization potentials from the *G*_0_*W*_0_*@*PBE calculation showed that the larger cluster has less variation, whereas the electron affinities of the series have an increasing trend. The ionization potentials from the *G*_0_*W*_0_ benchmark for the calcium cluster series have not yet been described in the literature.

## Introduction

Calcium metal has a closed shell structure and belongs to the group IIA alkaline-earth metals [1]. It has been widely used in carbon-chemical engineering (coating fullerene in hydrogen storage), optics, and materials science (as an ionic deposition) [2]. The clusters of calcium are essential because they bridge the atomic and bulk materials; therefore, revealing their transition from micro- to macroscopic characteristics is a significant undertaking [3]. Being a divalent metal, the size transition of calcium clusters occurs above the trimer cluster. Small calcium clusters (2–5) have been investigated using first-principles calculations incorporating the electron correlation effects [4–7]. In 3D cluster structures, it can be used in the manufacturing of coating materials, which is useful for high-capacity hydrogen storage [8]. As cluster size grows, the electronic configuration changes its semiconducting behavior from nonmetal to metallic due to the overlap between the s and p orbitals. Moreover, the geometry of clusters is related to their structural properties. Most of the studies on calcium clusters are limited to the dimer, where the binding energies and/or the ionization potentials (IPs) were determined spectroscopically [9–12]. In addition to the experiments, a few theoretical DFT studies on calcium clusters focus only on metallic behavior, vibrational frequency analysis, and thermodynamic properties [1,13–14]. However, there are no systematic studies on the electronic and structural properties of the series of calcium clusters.

Apart from some theoretical studies on calcium clusters, there are no systematic discussions on neutral cluster structural and electronic properties. Moreover, some studies have shown controversy in the binding energies of ground state clusters even for similar geometry and functionals used for calculation [1,13]. In addition, there are no reported *G*_0_*W*_0_ studies for Ca clusters, which in the past have provided better IP and *E*_gap_ for various systems. For small Ca clusters of up to 20 atoms, the structure, energies, and electronic structure were studied within the all-electron DFT approach. Our work aims to present the intricate characteristics of small Ca clusters by employing the DFT and state-of-the-art *G*_0_*W*_0_ approximation, which was recently used to predict the new ground-state structure of Be and Mg and successfully applied to obtain the correct IPs for these elemental clusters [15–16]. This comprehensive benchmark study will help to enhance our understanding of these fascinating nanostructures and lead to their real-world utilization in various technological advancements [2]. We used the DFT + *GW* scheme to investigate the electronic properties and structures of neutral Ca*_n_* (*n* = 2–20) clusters. From the DFT [17–18], one can obtain the accurate binding energies of the clusters, whereas the study of electronic properties from the *GW* approximation promises high accuracy in studying the excitation properties of the systems under consideration [19]. Despite the wide availability of theoretical and experimental work on Ca clusters, no *GW* studies have been performed on neutral calcium clusters to the best of our knowledge.

## Computational Method

Here, we employed particle swarm optimization (PSO) with CALYPSO code [20] interfaced with ABACUS code [21] to predict the neutral cluster of calcium (2–20) and local geometry optimization, respectively. The acquired structures were analyzed to determine among the low-energy isomers after running the calculation for 22 generations for calcium clusters (≈600 structures). Among the obtained 600 structures, the lowest four distinct isomers were selected for local geometry optimization for which GGA_PBE functional [22] was used. The PBE functional is generally computationally less demanding compared to other functionals, as it can be more efficient in terms of computational resources and time. The threshold for the force was set at 0.1 eV/Angstrom for better convergence, whereas the charge density difference tolerance, which is essential for convergence, was set at a value of 10^−9^. The ABACUS code employs the ONCV-type multi-projector pseudopotentials for the description of the core ions, which is used in our calculation as provided by the SG15 library [23]. We have therefore set the energy cutoff as 100 Ry for better accuracy. The double-ζ plus polarization (DZP) basis set was used in ABACUS calculations, which was tested against the triple-ζ plus double polarization (TZDP) to obtain the total energies of Ca_6_, Ca_7_, and Ca_8_. The convergence plot can be found in Supporting Information File 1, Figure S1. It is worth mentioning that our motivation for the selection of DZP is based on our previously reported data for DZP and TZDP basis sets, which were tested for various isomers of Be and Mg clusters. These results have shown that the basis sets are well converged and even DZP is sufficient to obtain reliable results [15].

To study the energetics of Ca*_n_* clusters, we used the *G*_0_*W*_0_ calculations in the FHI-aims code [24]. For the *G*_0_*W*_0_ calculations in the FHI-aims code, the NAO basis sets are employed with PBE functional to relax the structural geometry using the tier 4 and “tight” settings, providing a better description of IPs and HOMO–LUMO gaps of the molecules and clusters. In addition, the *G*_0_*W*_0_ calculations are performed to obtain *GW-*calculated eigenvalues using the two-pole fitting by setting 40 frequency points.

## Results and Discussion

### Structural geometry

**Figure 1:**
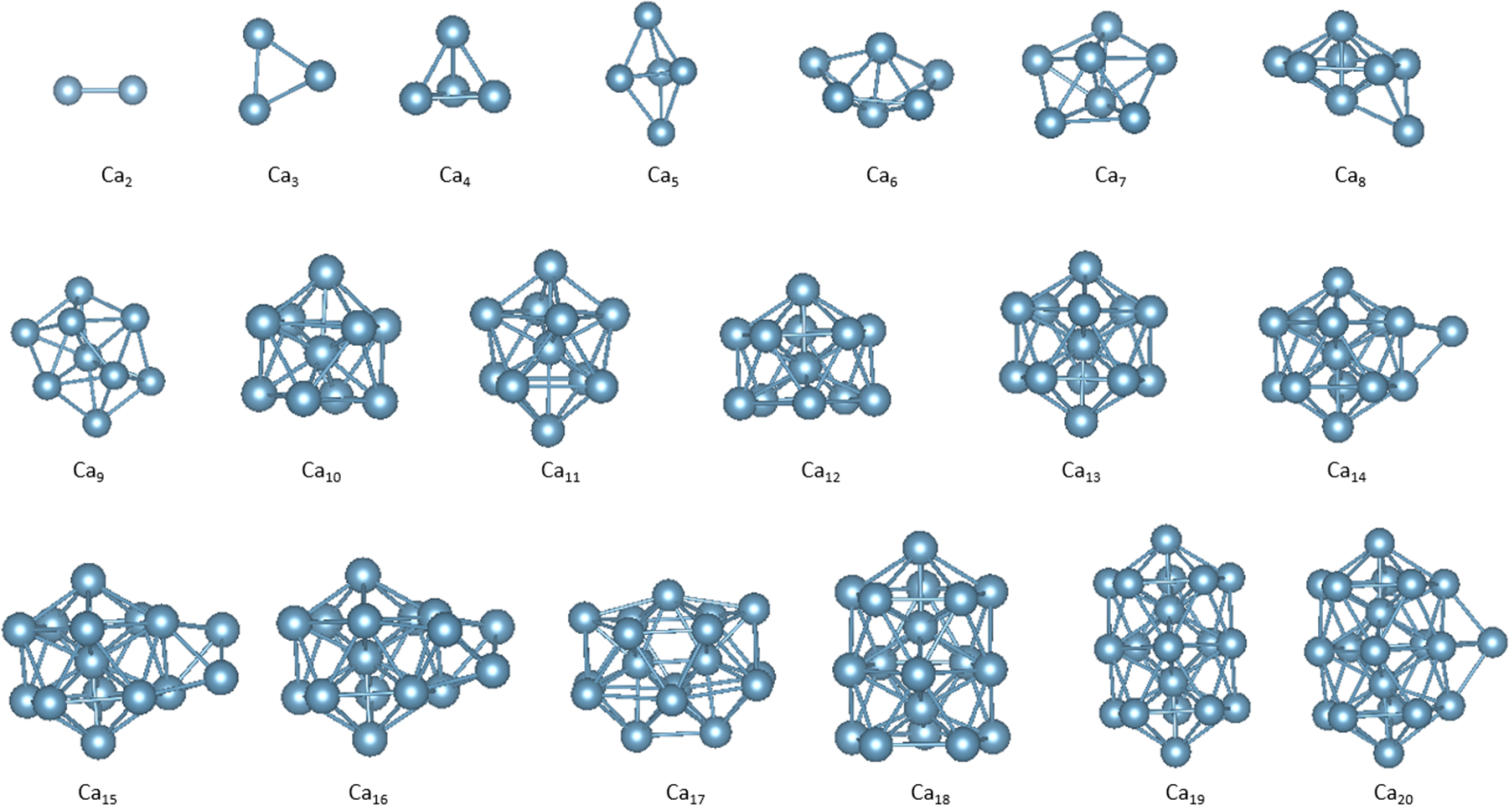
The ground-state structures of Ca clusters (*n* = 2–20).

The geometries of the ground-state structures of Ca clusters (2–20) were analyzed by the ABACUS software and are displayed in Figure 1.

**Figure 2:**
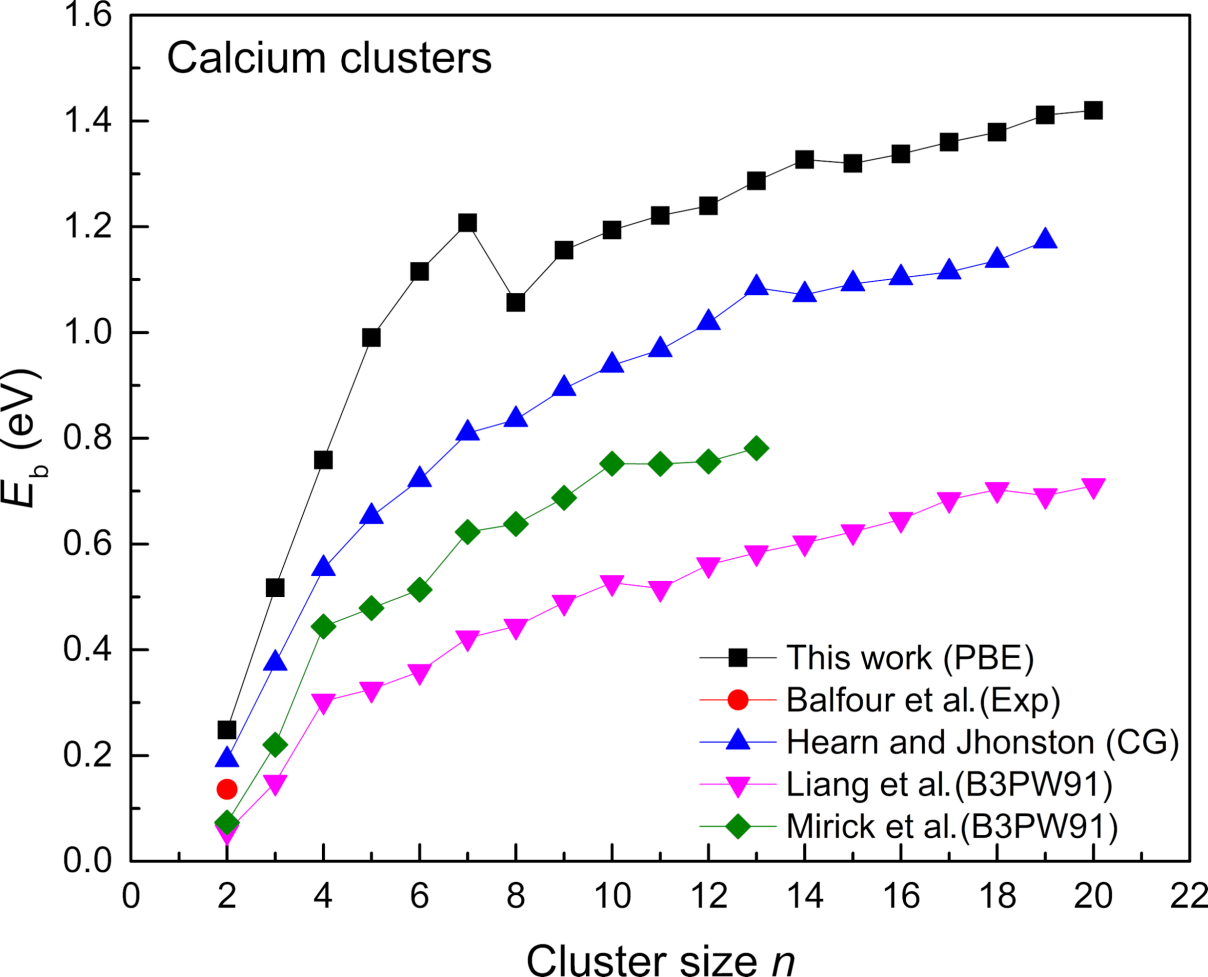
The binding energies of ground-state Ca clusters (*n* = 2–20) in comparison with reported data [1,11,13,17].

The geometrical structures predicted in this work, with a few exceptions similar to those obtained by the work of Liang et al. [13]. The trimer forms an equilateral triangle structure, whereas the three-dimensional configurations are more favorable for larger clusters. Figure 2 shows the binding energies of the cluster for the size range of 2–20 in comparison with reported data which is calculated from Equation 1:


[1] $$\frac{E_{\text{b}}}{n}=E_{\text{atom}}-\frac{E_{\text{tot}}}{\text{size}\left(n\right)}.$$

Here, *E*_tot_ is the total energy of the cluster after the relaxation step, *n* is the cluster size, and *E*_atom_ represents the free atom energy.

**Figure 3:**
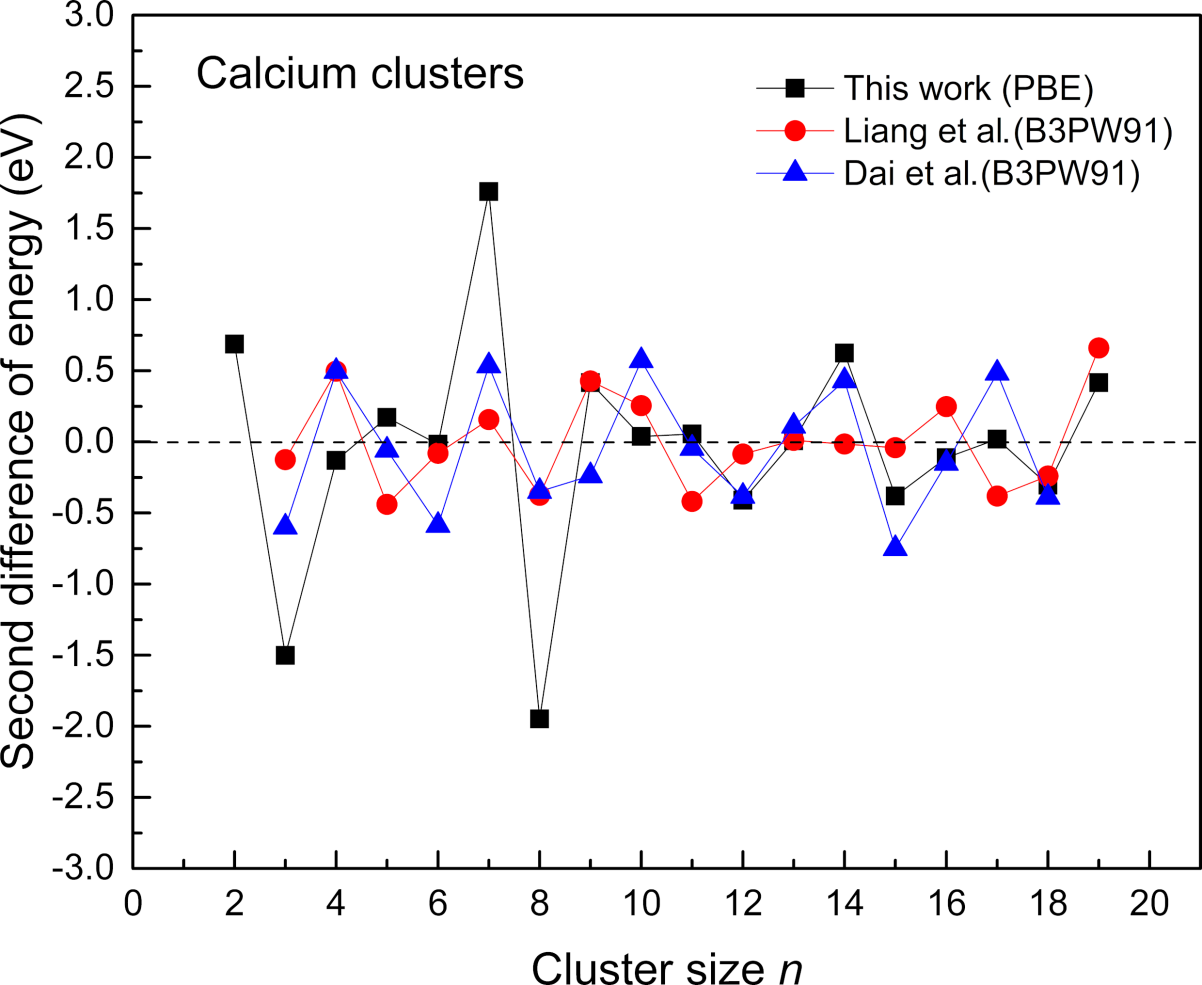
The second difference in energy for calcium ground-state clusters (*n* = 2–20) The data is plotted against the reported work of Liang et al. [13] and Dai and Blaisten-Barojas [26].

The experimental studies of calcium dimers showed that the ground state of Ca_2_ has a low binding energy of 0.14 eV. Our dimer results are also significantly closer to the reported experimental and theoretical work [13,17]. The binding energies also increase rapidly up to cluster size *n* = 7. A decrease in binding energy is seen at *n* = 8, likely due to the shell closing effect. Ca_7_ might represent such a stable configuration that an extra atom has to form Ca_8_ which could mean starting a new electronic shell and is less stable initially, resulting in a drop in binding energy. In addition, the structural geometry can play a crucial role in the stability of a cluster. Ca_8_ is a pentagonal bipyramid structure with a capped atom, which disrupts its symmetry from a perfect pentagonal bipyramid. This symmetry breaking can also be a possible reason, as it might generate a structure with increased surface strain or less favorable bonding environments. Apart from this, we can also analyze Ca_7_ whose binding energy is much higher in the series of clusters as shown in Figure 2. The higher binding energy depicts higher stability, which can be confirmed by the second difference analysis (see Figure 3). Another possible reason for the drop in binding energy from *n* = 7–8 can be linked to the shift from a more stable structure of Ca_7_ to a less stable structure of Ca_8_ (as the symmetry is disrupted by adding a capping atom). This observation was not seen in previous studies and could be due to the difference in the type of functional and code used in the calculation. The use of hybrid functionals generally improves accuracy for many systems, but it does not guarantee perfect results for all types of bonding or electron correlation effects. Overall, the accuracy of theoretically obtained structures is supported by the experimental data, which is lacking in this case. Apart from the accuracy of the functional, there can be a van der Waals interaction effect for clusters, which can be calculated by semi-empirical corrections added to the conventional density functional approximation and needs detailed assessment for small clusters (*n* = 2–10). In such cases, DFT-D methods can be used along with the standard DFT calculations.

By further increasing the cluster size, the binding energy smoothly increases again. The PBE results are plotted in comparison with the reported experimental work of Balfour et al. [11] and theoretical works [1,13,17]. Comparing the reported theoretical work, we can see some inconsistencies in the predicted binding energies at cluster size *n* = 8–11. In our work, the reported binding energies depict a sharp decrease in *E*_b,_ which is missing in other theoretical literature. In our case, the cluster size *n* = 8, which is a pentagonal bipyramid structure with an added atom to break its symmetry, can be a reason for a sudden drop in the cluster binding energy; however, a decrease or any change can also be attributed to the dispersion effect. Overall, the binding energies reported here are higher compared with all theoretically reported data. This difference can be due to two reasons. First is the use of a different functional and second are the basis sets and accuracy, which are different for the reported data compared with our work. In addition, van der Waals dispersion effects can also affect the binding energies of clusters, which need semi-empirical corrections [25]. We have also drawn a polynomial fit to calculate the convergent value of binding energy; however, the resulting value is 1.35 eV for binding energy per atom in Ca. This obtained value is small, as compared to the bulk value, which is 1.825 eV. In addition to the binding energies, the second difference in the energy analysis was carried out to analyze the stability of the geometrical structures, as shown in Figure 3.

It can be seen from the above figure that 2, 5, 7, 9, 10, 11, 14, 17, and 19 have positive values for the second energy difference. This indicates that these clusters are relatively stable among their neighboring clusters. In addition, it is seen that the clusters with sizes *n* = 4, 8, 12, 15, and 18 have negative second differences in energies, suggesting that these clusters are less stable among the series (see Figure 3). Moreover, clusters such as Ca_7_ and Ca_9_ have higher stability. The higher stability of these two clusters can be described by the ellipsoidal shell distortions that can occur in metal clusters [27]. A similar case was seen in our previous study of magnesium clusters, where the clusters with sizes *n* = 7, 15, and 17 showed relatively high stability from the second difference analysis [15]. In such cases, the predicted equilibrium shape of the cluster is ellipsoidal instead of spherical. It is also evident that the magic cluster Ca_10_ can be seen to have a lower positive second difference compared to other clusters in the series. The cluster exhibits a slightly distorted C_3v_ structure. In the case of Ca_10_, its deviation from a perfect tetrahedral structure could be a contributing factor to its relatively modest second difference in energy.

### Electronic properties

**Figure 4:**
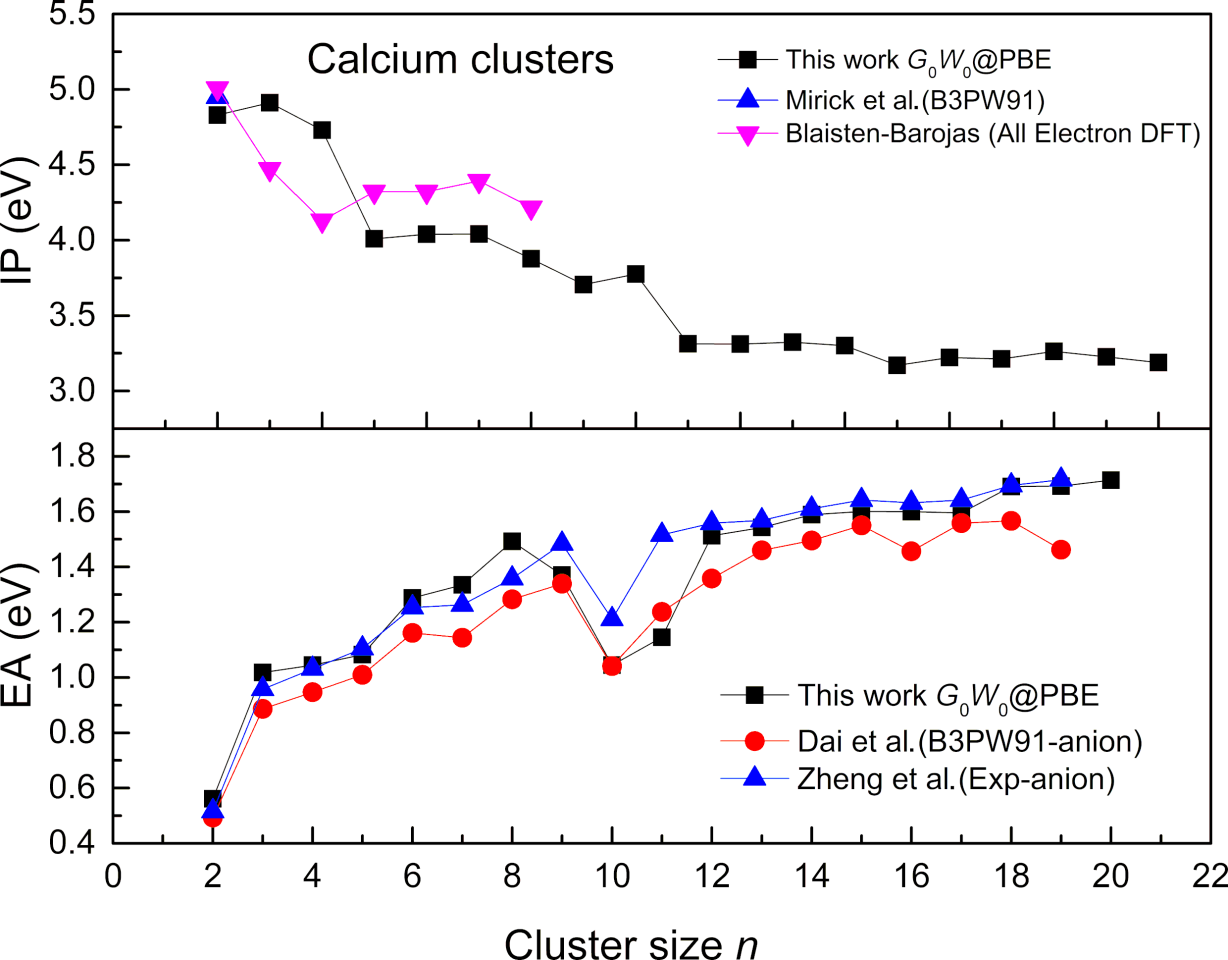
The IPs and EAs of calcium ground-state clusters (*n* = 2–20). The data is presented by *G*_0_*W*_0_ studies, compared with anionic experimental work [29] and theoretical reports [1,14,26].

The electronic properties of Ca clusters have been studied by the all-electron code FHI-aims, and the simulations are performed at the *G*_0_*W*_0_@PBE level. The IPs and EAs are presented in Figure 4 along with the reported data. Our dimer ionization potential, which is 4.83 eV for Ca clusters from *G*_0_*W*_0_@PBE is closer to the theoretical value of 4.95 eV [11]. In the upper panel of this figure, there is a decreasing pattern for the calcium cluster for ionization potentials. For the magic cluster Ca_10_, a larger ionization potential is obtained compared to the other neighboring clusters of the series. A swift fall in the ionization potential occurs for cluster Ca_11_, which shows the shell closing at *n* = 10. For larger clusters, the ionization potential curve becomes relatively smooth. Normally, as the cluster size increases, the IP tends to decrease. The smaller clusters tend to have higher IPs because electrons are more tightly bound. On the other hand, the larger clusters have more delocalized electrons, which results in lower IPs in these clusters. Clusters with lower IPs are generally more reactive, as they can more easily lose an electron. In our work, we have observed peaks at ≈4.83 and 4.90 eV. Such peaks suggest that these clusters are more stable than their neighboring clusters. The comparison with reported anionic clusters is not possible due to the unavailability of any data for EAs for neutral clusters. A sharp decrease in the EAs is obtained for Ca_10,_ whereas the ionization potential for this cluster is relatively high, which indicates it is a highly stable magic cluster. Our *G*_0_*W*_0_ IPs and EAs for the neutral cluster are reasonably close to the available data. Moreover, the EA shows an increasing trend, which means larger clusters have more electron affinity than smaller ones. For IPs, the Jellium even and odd oscillations except for a few clusters are not prominent for calcium clusters. This may suggest that the electron interactions in such clusters are more localized compared to the delocalized electron sea assumed in the Jellium model.

Another remarkable aspect of the cluster studies is to understand whether there is an underlying correlation between the ionization potential and electron affinities of the clusters. To study the correlation between the IPs and EA (as the function of cluster size), we applied a linear regression to analyze the quantitative estimation of the strength of the correlation for Ca clusters. For the binding energies versus the IPs, linear regression analysis returned the R^2^ value of 0.85. On the other hand, for binding energies versus EAs, we obtained the R^2^ value of 0.76. These results suggest there is a stronger correlation of binding energies *E*_b_ with IPs than EA. Moreover, the linear regression analysis for *E*_b_ versus *E*_gap_ yielded the R^2^ value of 0.89, which is the highest of all three. This suggests that the strongest correlation exists between the binding energies and *E*_gap_ for Ca clusters. In addition, we also plotted the second differences of the binding energies with the IP, EA, and *E*_gap_ which can be seen in Supporting Information File 1, Figure S2a–c. All three results in this figure show that the second difference between IPs and EAs closely follows *E*_b_ indicating a good correlation. Supporting Information File 1, Figure S2c indicates the relationship between the relative thermodynamic stability of the clusters and *E*_gap_ which is also called “the maximum hardness principle” [28]. From these three results we can see that the oscillatory pattern is moderated with the evolution of the cluster size. However, the majority of the values of IPs, EAs, and *E*_gap_ are positive and following closely which suggests a clear correlation.

## Conclusion

We presented a DFT and *G*_0_*W*_0_ combined approach to study the electronic and structural properties of neutral calcium clusters for a size range of *n* = 2–20. The initial geometries are obtained with the CALYPSO structure prediction algorithm, interfaced with the ABACUS code. The electronic properties, specifically the IPs and the EAs of Ca clusters, are calculated with the all-electron code FHI-aims with the *G*_0_*W*_0_ scheme. From the structural stability study, the second-difference analysis of the consecutive clusters for both binding energies demonstrated the structural stability of clusters such as Ca_2_, Ca_5,_ Ca_7_, Ca_9_, Ca_11_, Ca_14_, Ca_17_, and Ca_19_, in addition to Ca_10_, which is also a magic cluster. The ionization potentials for Ca clusters from the *G*_0_*W*_0_ scheme are reported for the first time. Moreover, the high ionization potentials are obtained by the state-of-the-art *G*_0_*W*_0_ approach at a particular cluster size; in this case, Ca_2_, Ca_3_, Ca_10_, Ca_14_, and Ca_18_ clusters, which suggests that to ionize these clusters, more energy is required. Hence, one can suggest that energetically, the most stable structures do not necessarily have the largest IPs or EAs. Additionally, clusters with a larger *E*_gap_ should have a higher abundance, which suggests they should be chemically more stable. In summary, predicting the ionization potentials adds to our understanding of the electronic structure and energetics of the calcium clusters. This benchmark may provide useful insights for future exploration of size-dependent properties and their potential applications, such as developing theoretical models and designing functional materials.

## Supporting Information

File 1 Additional experimental data.

## Data Availability

The data that support the findings of this study are available upon request from the corresponding author (Dr. Sunila Bakhsh).

## References

[1] Mirick J W, Chien C-H, Blaisten-Barojas E (2001). Phys Rev A.

[2] Bakhsh S (2023). Karbala Int J Mod Sci.

[3] de Heer W A (1993). Rev Mod Phys.

[4] Jones R O (1979). J Chem Phys.

[5] Pacchioni G, Koutecký J (1982). Chem Phys.

[6] Lee T J, Rendell A P, Taylor P R (1992). Theor Chim Acta.

[7] Bagus P S, Neun C J, Bauschlicher C W (1985). Surf Sci.

[8] Yoon M, Yang S, Hicke C, Wang E, Geohegan D, Zhang Z (2008). Phys Rev Lett.

[9] Bondybey V E, English J H (1984). Chem Phys Lett.

[10] Hansen C S, Calaway W F, King B V, Pellin M J (1998). Surf Sci.

[11] Balfour W J, Whitlock R F (1975). Can J Phys.

[12] Allard O, Samuelis C, Pashov A, Knöckel H, Tiemann E (2003). Eur Phys J D.

[13] Liang X, Huang X, Su Y, Zhao J (2015). Chem Phys Lett.

[14] Blaisten-Barojas E, Chien C-H, Pederson M R, Mirick J W (2004). Chem Phys Lett.

[15] Bakhsh S, Liu X, Wang Y, He L, Ren X (2021). J Phys Chem A.

[16] Bakhsh S, Khalid M, Aslam S, Sohail M, Iqbal M A, Ikram M, Morsy K (2024). Beilstein J Nanotechnol.

[17] Hearn J E, Johnston R L (1997). J Chem Phys.

[18] Iqbal M A, Ashraf N, Shahid W, Afzal D, Idrees F, Ahmad R (2022). Fundamentals of Density Functional Theory: Recent Developments, Challenges and Future Horizons. Density Functional Theory - Recent Advances, New Perspectives and Applications.

[19] van Setten M J, Caruso F, Sharifzadeh S, Ren X, Scheffler M, Liu F, Lischner J, Lin L, Deslippe J R, Louie S G (2015). J Chem Theory Comput.

[20] Wang Y, Lv J, Zhu L, Ma Y (2012). Comput Phys Commun.

[21] Li P, Liu X, Chen M, Lin P, Ren X, Lin L, Yang C, He L (2016). Comput Mater Sci.

[22] Perdew J P, Burke K, Ernzerhof M (1996). Phys Rev Lett.

[23] (2024). Quantum-simulation.

[24] Blum V, Gehrke R, Hanke F, Havu P, Havu V, Ren X, Reuter K, Scheffler M (2009). Comput Phys Commun.

[25] Grimme S (2006). J Comput Chem.

[26] Dai Y, Blaisten-Barojas E (2008). J Phys Chem A.

[27] Clemenger K (1985). Phys Rev B.

[28] Parr R G, Chattaraj P K (1991). J Am Chem Soc.

[29] Zheng W (2005). Negative Ion Photoelectron Spectroscopy of Metal Clusters, Metal-Organic Clusters, Metal Oxides, and Metal-Doped Silicon Clusters.

