# Supplementary material for: Can neutral clusters: a two-step G0W0 and DFT benchmark
Source: Beilstein J Nanotechnol. 2024 Aug 8;15:1010–6. doi: 10.3762/bjnano.15.82 (PMC11318633; doi:10.3762/bjnano.15.82)
Supplement: File 1 — Additional experimental data. [file Beilstein_J_Nanotechnol-15-1010-s001.pdf]

BEILSTEIN JOURNAL OF NANOTECHNOLOGY

Supporting Information

for

Ca_n neutral clusters: a two-step G_0W_0 and DFT benchmark

Sunila Bakhsh, Sameen Aslam, Muhammad Khalid, Muhammad Sohail, Sundas Zafar, Sumayya Abdul Wadood, Kareem Morsy and Muhammad Aamir Iqbal

Beilstein J. Nanotechnol. **2024,** *15,* 1010–1016. doi:10.3762/bjnano.15.82

Additional experimental data

License and Terms: This is a supporting information file under the terms of the Creative Commons Attribution License (https://creativecommons.org/licenses/by/4.0). Please note that the reuse, redistribution and reproduction in particular requires that the author(s) and source are credited and that individual graphics may be subject to special legal provisions.
The license is subject to the *Beilstein Journal of Nanotechnology* terms and conditions: (https://www.beilstein-journals.org/bjnano/terms)

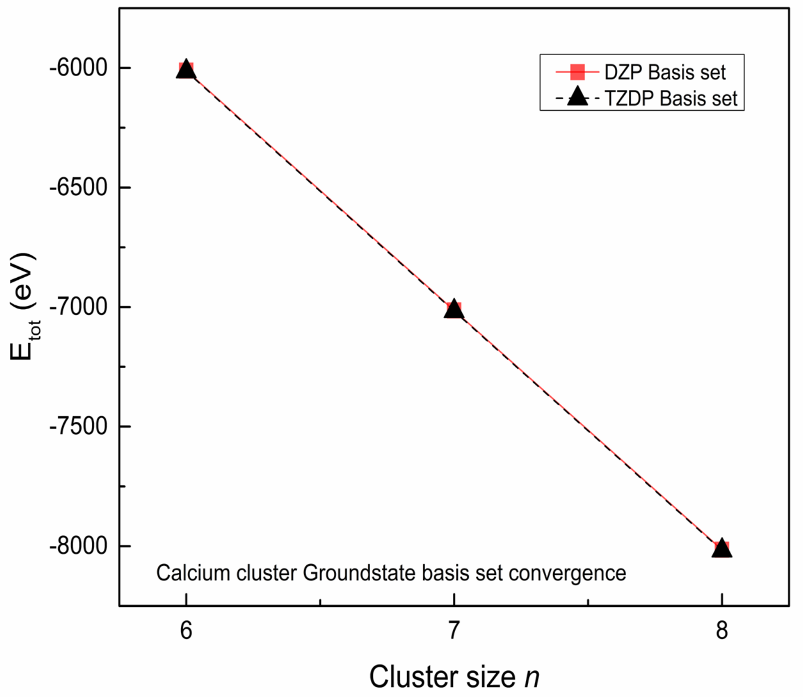

Figure S1: The DZP and TZDP basis set convergence results of the total energy of ground state calcium clusters for size n = 6–8.

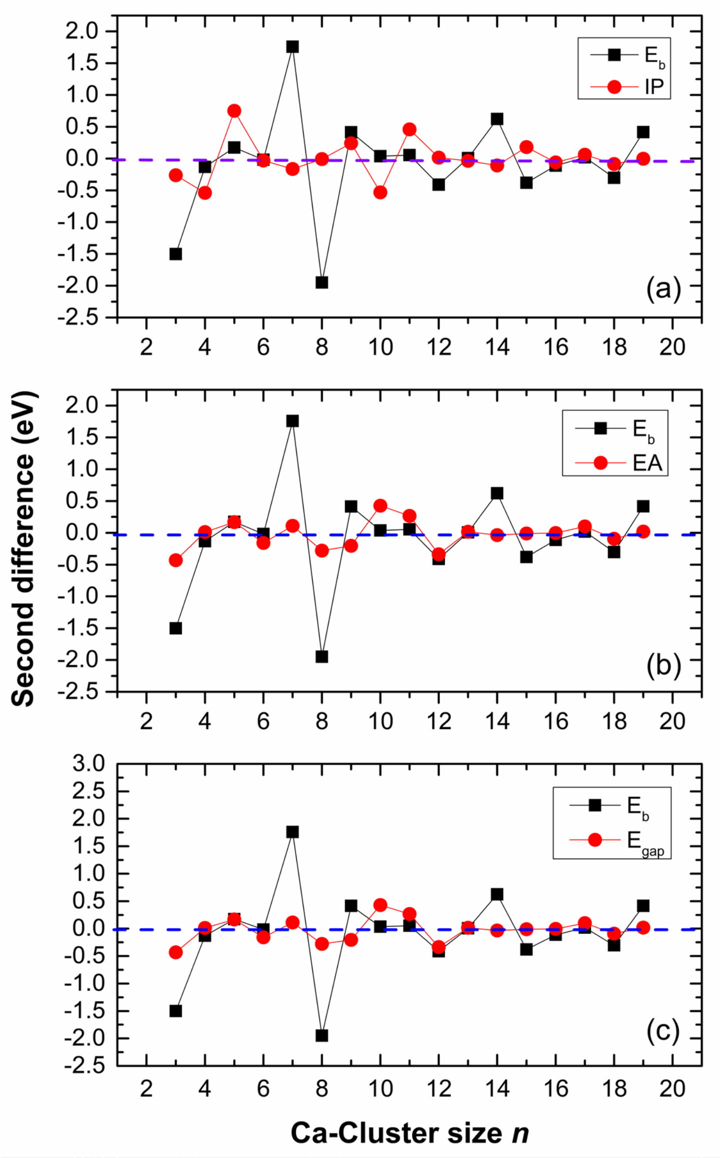

Figure S2: The second difference of energy for E_b versus a) IP, b) EA and, c) E_{gap}.

The ground state geometry files are provided in XYZ format below:

3:

atom 10.833176065055 10.317750000000 12.144787498620 Ca

atom 9.664883925490 12.180606632466 9.137200534686 Ca

atom 9.664883925490 8.454893367535 9.137200534685 Ca

4:

atom 8.960168136790 11.532807783015 8.960106654168 Ca

atom 11.532744417006 8.960241207357 8.960241206972 Ca

atom 11.532724238207 11.532444355830 11.532444355416 Ca

atom 8.960168135992 8.960106653797 11.532807783443 Ca

5:

atom 5.580859737505 5.504299999975 8.499691822744 Ca

atom 5.580859281894 5.504299999972 1.999348074752 Ca

atom 3.532854309305 5.504300000007 5.249521551292 Ca

atom 6.608578748135 3.720961914433 5.249521770605 Ca

atom 6.608578748145 7.287638085539 5.249521770604 Ca

6:

atom 6.097958149777 1.809137534915 5.508531960574 Ca

atom 5.028515091569 5.066023558045 7.266071046153 Ca

atom 5.023809343590 5.065969641420 3.754705599254 Ca

atom 5.518779752617 8.312453441437 5.509800508595 Ca

atom 8.290376714087 5.388440321591 5.506494041479 Ca

atom 1.863344384364 6.344698220586 5.514633393965 Ca

7:

atom 7.212460133705 8.065481879939 5.462173039224 Ca

atom 5.392512056436 1.804576195115 5.462070267524 Ca

atom 5.393582829756 5.165406952071 3.743545853803 Ca

atom 8.651208030106 4.443866790440 5.463617771039 Ca

atom 5.392046583451 5.165525790206 7.180385296798 Ca
atom 2.134468752206 4.443267367219 5.460794610913 Ca
atom 3.571795414352 8.064615271018 5.461401060691 Ca

8:

atom 13.046813582334 9.941752879045 13.308778267410 Ca
atom 11.564029619015 10.361236652804 9.971060865454 Ca
atom 15.274755316530 10.389943950399 10.418541469294 Ca
atom 13.231557630514 13.144047286364 11.718197588257 Ca
atom 12.810226348983 12.856075908408 15.421607725608 Ca
atom 10.693425097439 9.897324924124 16.175893584205 Ca
atom 16.094001522032 11.957250827966 13.712052307584 Ca
atom 10.003112123166 11.867326085884 13.102865102252 Ca

9:

atom 10.385602086230 9.961683368563 11.783025201576 Ca
atom 7.257640539800 11.782479099888 12.228007507681 Ca
atom 7.261688881845 8.224259301521 11.057752872286 Ca
atom 13.511478371425 8.226374931814 11.062706934255 Ca
atom 13.511566143155 11.784928622459 12.232393846185 Ca
atom 10.385032705395 13.464983898157 10.865410510420 Ca
atom 8.525529269520 10.929373016834 8.836969317548 Ca
atom 10.388337627178 7.687420171827 8.962481769784 Ca
atom 12.249271095435 10.930455582960 8.839795590249 Ca

10:

atom 10.492678464022 10.492673809227 10.650711963591 Ca
atom 8.354080700416 8.354874422192 9.404701663324 Ca
atom 12.631196526045 12.630566357695 9.404676005979 Ca
atom 12.631058023249 8.354686888915 9.404619308014 Ca
atom 8.353944660128 12.630375835397 9.404772051386 Ca
atom 10.492632112017 10.492757247144 7.158166156144 Ca

atom	10.492701077203	7.772781161367	12.594428408044	Ca
atom	10.492706875667	13.212528458539	12.594409791118	Ca
atom	13.211342974498	10.492630837141	12.595557184226	Ca
atom	7.774158586794	10.492624982345	12.595809015216	Ca

11:

atom	10.408476989859	10.408155379662	10.800215055829	Ca
atom	10.408282332533	10.408450532312	7.015359495803	Ca
atom	10.407945656452	10.408425944249	14.584166408307	Ca
atom	11.535828914363	13.129788950354	9.222481729910	Ca
atom	9.280763624795	7.686886271543	9.221200744935	Ca
atom	7.686933716995	11.535874494659	9.221717060702	Ca
atom	13.129945109060	9.280692813738	9.222275853011	Ca
atom	11.535707524921	7.686462671220	12.377830438218	Ca
atom	9.280642169922	13.129893766038	12.377668165833	Ca
atom	13.129785730398	11.535793097787	12.378580451431	Ca
atom	7.686438230703	9.280742408389	12.377070593015	Ca

12:

atom	12.844523371080	13.008637884103	13.441474630934	Ca
atom	10.029882734546	14.881969279538	14.725468446801	Ca
atom	10.095641300049	11.033412766620	14.716078455899	Ca
atom	15.570100078384	14.960139914366	12.091279335753	Ca
atom	15.635187613356	11.157408245335	12.082662585001	Ca
atom	11.950474408905	16.073961522385	11.747856885505	Ca
atom	12.056079050039	9.921185589700	11.733181112568	Ca
atom	15.952639666507	13.057563990649	15.287627459241	Ca
atom	9.778684806327	12.959531387243	11.528605941206	Ca
atom	13.651311541214	16.134983649016	15.075662094827	Ca
atom	13.757659329674	9.903556225262	15.061280080327	Ca
atom	13.188185931839	13.022473005790	9.852864023953	Ca

13:

atom	10.627935192471	8.692806483707	7.526735298034	Ca
atom	10.626947808193	12.516761584926	7.526550640790	Ca
atom	7.564140546725	10.605162770093	8.724522481821	Ca
atom	8.718086605344	7.511675757299	10.619769816321	Ca
atom	12.493775609911	7.511457102698	10.590620484595	Ca
atom	13.672245676899	10.604678179572	8.678734505620	Ca
atom	12.491618377452	13.697653411904	10.589967677585	Ca
atom	8.716421167135	13.698587681745	10.619230922662	Ca
atom	10.582238425078	12.516964293131	13.682874610973	Ca
atom	13.645391566963	10.604727881406	12.484829138088	Ca
atom	10.583331678708	8.693141828895	13.683417249108	Ca
atom	7.537362761275	10.605256730237	12.531406028053	Ca
atom	10.604416681839	10.604826294387	10.604829048316	Ca

14:

atom	10.868914718045	8.559639529307	13.501584747872	Ca
atom	13.936744451979	10.437567350142	12.307025561650	Ca
atom	12.736562311603	13.499983240111	10.424118645657	Ca
atom	8.902909342192	13.535212616397	10.506230626899	Ca
atom	7.759057282117	10.509883895299	12.391527874367	Ca
atom	10.785681457256	12.393643870479	13.534429057397	Ca
atom	8.988736925728	7.357394200132	10.438368309264	Ca
atom	12.795542450236	7.339542311524	10.412603112976	Ca
atom	13.955270863112	10.408824015884	8.500180965869	Ca
atom	7.794841367032	10.423808550946	8.557694813407	Ca
atom	10.885275591936	8.497067732685	7.341113856845	Ca
atom	10.858510123594	12.303702447135	7.356766701895	Ca
atom	10.890410606401	10.404039104016	10.404558769454	Ca
atom	7.409340733765	13.888118804976	13.882224625403	Ca

15:

atom	10.153181848056	9.868258478728	15.200282044134	Ca
atom	10.378523184025	10.186428408192	11.624591585208	Ca
atom	10.668581998706	10.595328143126	7.898208723423	Ca
atom	13.599032155455	9.474631992648	9.933067807942	Ca
atom	12.440323579120	13.016071358106	10.252878978951	Ca
atom	8.713256425984	13.039653477156	9.944049438439	Ca
atom	7.462380147322	9.400437596501	9.700412028454	Ca
atom	10.514537679519	7.173599029486	9.693815514140	Ca
atom	7.235453272412	11.083703041656	13.065740993216	Ca
atom	8.387799065431	7.454032742328	12.852808637363	Ca
atom	12.196217088602	7.464352202610	13.055850283981	Ca
atom	13.340062662394	11.034849108918	13.449683222189	Ca
atom	10.288080947330	13.262410051306	13.455969313503	Ca
atom	13.977449281801	11.961598568626	7.092225432485	Ca
atom	10.956872439806	14.165309984603	7.099366659586	Ca

16:

atom	10.503291057569	9.800978602681	9.849876747700	Ca
atom	10.499562510900	14.734442806382	13.275311174071	Ca
atom	10.502971597941	7.409152257998	12.534452086284	Ca
atom	13.587679032393	9.619744204717	11.859300100967	Ca
atom	12.381676216878	12.948747876455	10.569860930482	Ca
atom	8.621711246024	12.947100068261	10.567928785476	Ca
atom	7.417260461940	9.617149172765	11.855884716712	Ca
atom	8.590042962381	6.829009109280	9.338756458615	Ca
atom	12.419965090538	6.830627073154	9.340824322225	Ca
atom	10.505713235526	8.541222520068	6.494642280230	Ca
atom	7.439065665442	10.202656233077	8.048577057339	Ca
atom	10.503698232200	12.255667701481	7.246374526773	Ca
atom	13.569466545057	10.205372907959	8.051870874808	Ca
atom	7.303201099162	12.764994508179	14.032085166816	Ca

atom 10.501011811166 11.137670948368 13.354700218413 Ca
atom 13.696883234884 12.767910349160 14.035325753126 Ca

17:
atom 14.711761913330 15.767310590507 18.386199186083 Ca
atom 14.050246086204 14.482517387354 10.149391211346 Ca
atom 16.568308971913 12.932898364401 16.683272747110 Ca
atom 10.679302224127 12.851614946375 10.747347534786 Ca
atom 16.602198845593 13.409840246899 12.847605921756 Ca
atom 9.798311546043 13.474755002311 14.382504499391 Ca
atom 13.054661388862 12.934198786344 13.152927155733 Ca
atom 14.418440011500 15.206202804042 15.064501497540 Ca
atom 15.634890102366 17.121723402885 12.370603925085 Ca
atom 12.728648030514 12.492576577994 16.703660192636 Ca
atom 11.382892589578 16.114009045982 16.603709169611 Ca
atom 14.110818379566 10.746826973331 10.800266809785 Ca
atom 11.878320248055 16.308154104149 12.775035535472 Ca
atom 14.983776144150 10.293549223422 14.462574383929 Ca
atom 11.297044266213 10.079999741555 13.602243008329 Ca
atom 14.093515069088 18.539815230953 15.531902208652 Ca
atom 17.526300400837 16.433495803608 15.584844876745 Ca

18:
atom 10.744530499686 10.828628977772 9.408808049280 Ca
atom 10.747079991013 10.832057516801 13.016817716531 Ca
atom 9.738836671849 7.712640303378 11.214371831117 Ca
atom 13.399975067164 8.908798268889 11.210525054434 Ca
atom 13.393589447549 12.760348826184 11.206742239561 Ca
atom 9.728604879026 13.944848269910 11.208351540491 Ca
atom 7.469440956772 10.825139350621 11.212673759752 Ca
atom 13.983702927128 10.832171823349 7.910570410907 Ca
atom 11.740346344512 13.910194219232 7.909081939300 Ca

atom	8.119561668507	12.728000177879	7.912723867308	Ca
atom	8.125566681751	8.918547735412	7.916253588025	Ca
atom	11.749685375385	7.747448862317	7.914858331782	Ca
atom	14.045770288387	10.839051183072	14.406697132227	Ca
atom	11.761453766213	13.970231435531	14.405950428418	Ca
atom	8.077451042031	12.766512006736	14.409587125223	Ca
atom	8.083734261430	8.891420210219	14.413221960418	Ca
atom	11.772004066392	7.700225010729	14.412308754110	Ca
atom	10.742065751224	10.825535821969	5.861570175080	Ca

19:

atom	11.264468743402	13.787254725616	18.561825816611	Ca
atom	15.355698896911	15.489437980506	10.412240560288	Ca
atom	17.099103777981	14.291360761239	16.248958379114	Ca
atom	19.038273831058	11.948376908596	13.863170864053	Ca
atom	14.190802460169	16.213485717315	17.906421825006	Ca
atom	18.319747333552	15.537912237054	12.806810694472	Ca
atom	14.909463402401	12.624409744189	18.963222954407	Ca
atom	18.009931915343	12.685060650092	10.228923106621	Ca
atom	13.486893183610	13.431771461752	15.799471635695	Ca
atom	15.771730595778	13.057591160608	12.976524877909	Ca
atom	10.113837715629	12.544796166850	15.090273549549	Ca
atom	12.988901339152	10.594336699520	13.410665735977	Ca
atom	11.226770169179	16.165133404337	15.512047887436	Ca
atom	14.804533741143	16.495508766467	14.099018768702	Ca
atom	15.976767263354	10.641979903468	15.824100401673	Ca
atom	16.519137315381	9.681589996598	12.124000774716	Ca
atom	12.390164230793	10.357717921678	17.223236420553	Ca
atom	14.242995324067	11.868578874130	9.990375509771	Ca
atom	12.266164157089	14.210945045066	12.345895607450	Ca

20:

atom	13.566613688294	13.182969684549	15.620392122531	Ca
atom	12.721711689435	13.842806994966	9.757051303869	Ca
atom	13.782180630384	15.460487483155	12.833239743900	Ca
atom	10.921466499385	12.996853295842	12.932211753129	Ca
atom	12.351961017458	9.931544544609	14.543917123455	Ca
atom	16.019281688226	10.590907779498	15.629079389843	Ca
atom	16.531456211284	17.715830490674	12.917468520835	Ca
atom	12.950199971645	18.841648936887	13.560115361675	Ca
atom	13.956986093548	17.617701320166	10.029983480600	Ca
atom	10.639133980074	16.574403531990	11.563807662520	Ca
atom	11.745000655012	10.193378189468	10.705439758084	Ca
atom	14.642715715292	11.827326487268	12.263979252262	Ca
atom	16.413319489292	14.759094619202	10.527399629413	Ca
atom	13.319213033802	10.869384950459	18.290127369826	Ca
atom	16.943306148362	14.132917581570	14.325635928519	Ca
atom	14.764613118916	16.654928593568	16.217103662556	Ca
atom	11.089069937369	15.972843439964	15.376314815104	Ca
atom	10.245118193312	12.405834444487	16.665760579728	Ca
atom	16.133850163347	13.514477618907	18.060093245789	Ca
atom	12.552490303573	14.639831481793	18.703084567287	Ca
